# Errata for Issue 110 (3)

**DOI:** 10.5195/jmla.2022.1671

**Published:** 2022-10-01

**Authors:** Katelyn Arnold

“Rigor and reproducibility instruction in academic medical libraries,” 2022;110(3):281-93

The PDF and metadata as published contained an incorrect author last name. The correct author name is Tisha Mentnech.

“Determining COVID-19's impact on an academic medical library's literature search service,” 2022;110(3):316-22

The PDF as published contained an incorrect author last name. The correct author name is David Petersen.

“In Memoriam: Virginia M. Bowden,” 2022;110(3):381-82

The article was published with an incorrect HTML title and incorrect author affiliation information. The correct affiliation for Janna C. Lawrence is Director.

